# Evaluation of Implant Collar Surfaces for Marginal Bone Loss: A Systematic Review and Meta-Analysis

**DOI:** 10.1155/2016/4987526

**Published:** 2016-07-14

**Authors:** Roodabeh Koodaryan, Ali Hafezeqoran

**Affiliations:** Department of Prosthodontics, Faculty of Dentistry, University of Medical Sciences, Tabriz 5166614711, Iran

## Abstract

*Background*. It is important to understand the influence of different collar designs on peri-implant marginal bone loss, especially in the critical area.* Objectives*. The purpose of the present systematic review and meta-analysis was to compare dental implants with different collar surfaces, evaluating marginal bone loss and survival rates of implants.* Methods*. Eligibility criteria included clinical human studies, randomized controlled trials, and prospective and retrospective studies, which evaluated dental implants with different collar surface in the same study.* Results*. Twelve articles were included, with a total of 492 machined, 319 rough-surfaced, and 352 rough-surfaced microthreaded neck implants. There was less marginal bone loss at implants with rough-surfaced and rough-surfaced microthreaded neck than at machined-neck implants (difference in means: 0.321, 95% CI: 0.149 to 0.493; *p* < 0.01).* Conclusion*. Rough and rough-surfaced microthreaded implants are considered a predictable treatment for preserving early marginal bone loss.

## 1. Introduction

The long-term clinical and aesthetic outcome of implant-supported restoration depends on preservation of both soft and hard tissues around implant [1–3]; thus the overall amount of crestal bone loss may influence the clinical success. Initial breakdown of peri-implant bone takes place in the most coronal portion of the bone-implant interface [1]. Bone resorption of 1.5 to 2 mm is observed during the first year of function and is generally considered a normal physiologic process. Successive annual bone loss of 0.2 mm occurs in subsequent years [4–6].

Many factors have been proposed to contribute to the marginal bone loss (MBL) around an implant. Factors such as unfavorable stress distribution, surgical trauma, implant-abutment microgap, and bacterial infiltration result in apical migration of the biologic width; thus the bone is protected from further irritation [7–10]. Implant neck design and surface characterization have been associated with reduced marginal bone loss [1, 11, 12]; this has led to the development of implants with new collar configuration and topographic modification in order to improve the soft and hard tissue osseointegration. Up to date, there is no consensus in the literature relative to the effectiveness of these configurations and their influence on the MBL. Implants with a shorter polished smooth collar have proven to be more effective in decreasing MBL [13]. Likewise, implants with coronal retentive grooves may provide more stable peri-implant bone levels [14–16]. Although the amount of MBL after functional loading was not significant with regard to rough and microthreaded configuration, the polished collar showed the highest amount of crestal bone loss in any follow-up periods [15]. Unfortunately, the available data are not sufficient to sustain a conclusion with regard to the neck configurations.

The present systematic review and meta-analysis were conducted to evaluate the MBL around implants with different collar surfaces. The null hypotheses are as follows: (1) there are no differences in marginal bone loss in patients who received dental implants with different collar surface characterizations and (2) there are no differences among dental implants with different collar characterizations with regard to survival rates of implants.

## 2. Methods

This systematic review adheres to the criteria of the PRISMA statement [17]. Electronic searches without time restrictions were performed in the PubMed, Embase, and The Cochrane Library databases for relevant publications until 15 July 2016. Search terms used in this study were dental implant, oral implant, neck, design, bone remodeling, and marginal bone loss connected with OR and AND.

Authors also manually searched the literature for relevant publications in* British Journal of Oral and Maxillofacial Surgery, Clinical Implant Dentistry and Related Research, Clinical Oral Implants Research, European Journal of Oral Implantology, Implant Dentistry, International Journal of Oral and Maxillofacial Implants, International Journal of Oral and Maxillofacial Surgery, International Journal of Periodontics and Restorative Dentistry, International Journal of Prosthodontics, Journal of Clinical Periodontology, Journal of Dental Research, Journal of Dentistry, Journal of Oral Implantology, Journal of Craniofacial Surgery, Journal of Cranio-Maxillofacial Surgery, Journal of Maxillofacial and Oral Surgery, Journal of Oral and Maxillofacial Surgery, *and* Journal of Periodontology*.

### 2.1. Eligibility Criteria

The studies were included if they met the following inclusion criteria: (1) randomized clinical trials (RCTs), (2) retrospective and prospective studies, (3) comparing different collar surfaces, (4) follow-up periods of longer than 1 year, and (5) published in English.

Exclusion criteria were (1) case reports, (2) computational studies, (3) animal studies, (4) in vitro studies, (5) studies that evaluated only one type of collar design, and (6) review papers.

Based on population, intervention, control, and outcome (PICO) criteria the focused question was “what is the best implant collar configuration for preservation of MBL?” and the population was the patients undergoing implant-prosthetic rehabilitation with machined, rough, or rough-surfaced microthreaded neck configurations. The two outcomes evaluated were the survival rates and MBL of implants.

### 2.2. Study Selection

The titles were screened independently by the two reviewers. Abstracts of studies were inspected and those appearing to meet the inclusion were retrieved. Also, all reference lists of the selected studies and relevant reviews were scanned to identify articles that have been missed in database searches. Disagreements were settled by discussion between the authors until a consensus was achieved.

### 2.3. Quality Assessment

All studies were assessed for quality depending on whether they met all the quality criteria or if one or more criteria were partially met or not met using the Jadad scoring system [18] which ranges from 0 to 5. Studies with a Jadad score of 3 or higher were considered of high quality.

### 2.4. Summary Measures

The meta-analysis was based on the Mantel-Haenszel and inverse variance methods. Survival rates of implants were the dichotomous outcome measure expressed in risk ratio (RR) and marginal bone loss and the continuous outcome measure expressed in mean difference (MD), both with a 95% confidence interval (CI). The RR and MD values were considered significant when *p* < 0.05. The data were analyzed using comprehensive meta-analysis software (CMA 2.0) (BioStat Inc., Englewood, New Jersey, USA).

## 3. Results

### 3.1. Literature Search

**Figure 1:**
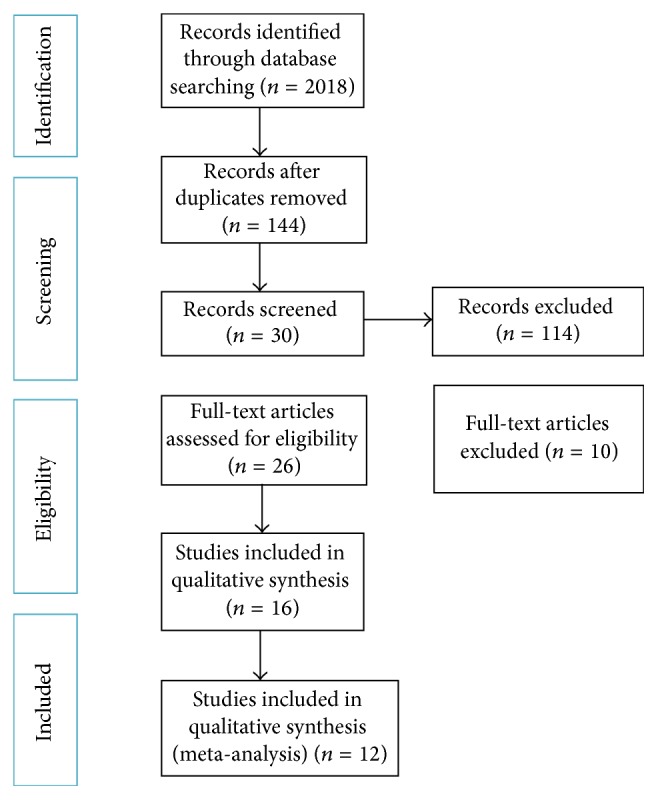
Diagram of the search strategy.

The search in the databases retrieved 2018 references, including 1778 from PubMed/MEDLINE, 229 from Embase, and 11 from The Cochrane Library. The identification and removal of duplicate references and application of the inclusion/exclusion criteria yielded 19 publications (14 cohorts, 2 retrospective studies, and 3 randomized controlled trials (RCT)) for further eligibility assessment (Figure 1). After qualitative assessment of the selected studies and reading the full texts of these publications, 12 studies [3, 15, 19–29] remained for inclusion in the quantitative meta-analysis (Tables 1 and 2).

### 3.2. Description of the Studies

A total of 12 studies were included in this quantitative meta-analysis which were published from 1998 to 2015. A total of 1163 implants and 930 patients were evaluated, and of these, 492 implants were machined, 319 rough-surfaced, and 352 rough-surfaced microthreaded. The follow-up periods were between 1 and 10 years. Eight of the selected studies [3, 15, 21–25, 27] evaluated the survival rates in relation to the type of collar surface characterization. Radiographic evaluation of MBL was performed by means of periapical radiographs in 9 studies [3, 15, 20–24, 27, 28], panoramic radiographs in two studies [19, 25], and maxillofacial CT in one study [26]. Rough and machined collars were considered in 7 studies [15, 20, 21, 23, 24, 26, 28]; while machined and microthreaded neck implants were installed in seven studies [3, 15, 19, 21, 22, 24, 25]. Only four studies compared microthreaded collar with roughened neck surfaces [15, 21, 24, 27].

The RCT study by den Hartog et al. [3] found a significantly greater marginal bone loss around smooth collars (1.19 ± 0.82 mm) compared with rough-surfaced neck implants (0.90 ± 0.57 mm) after 18 months of implant placement. One smooth-necked implant was lost 5 months after implant placement; thus, the survival rate was 96.8% at 18 months after implant placement compared with 100% of rough-surfaced collar. Nickenig et al. [19] determined marginal bone level changes around 70 rough-surfaced microthreaded and 63 machined-neck implants at six time points of implant placement, with a median follow-up time of 5.2 years. The two implant types revealed significant marginal bone level changes. The machined-neck implants were associated with a mean bone loss of 0.8 mm after six months of loading, 1.1 mm at two years' follow-up, 1.3 mm at three years' follow-up, and 1.4 mm at five years' follow-up, while the rough-surfaced microthreaded implants showed a mean crestal bone loss of 0.4 after six months of loading, 0.5 mm at two years' follow-up, 0.6 mm at three years' follow-up, and 0.7 mm at five years' follow-up. A 10-year retrospective study of 400 patients receiving 1244 implants by Sánchez-Siles and colleagues [20] assessed radiographic bone loss around implants with or without smooth collar designs. It was observed that smooth-necked implants had significantly lower amounts of marginal bone loss (1.18 ± 1.39 mm) compared with rough-surfaced implants (2.41 ± 1.35 mm) after 10 years of function (*p* < 0.001). Piao et al. [21] compared three different implant systems with a machined, rough, and rough-surfaced microthreaded neck in relation to marginal bone loss and detected significant differences (*p* < 0.0001). Implants with the rough-surfaced microthreaded collar surfaces had the least amount of bone loss (0.42 ± 0.27 mm) while the machined surface had the greatest amount (0.89 ± 0.41 mm) after one year of loading. In a RCT study by Peñarrocha-Diago et al. [22] MBL was evaluated around 69 dental implants with machined surface collar, external connection, and without platform switching and 72 implants with rough-surfaced microthreaded collar, internal connection, and with platform switching. MBL changes for machined and microthreaded implants were 0.38 ± 0.51 mm and 0.12 ± 0.17 mm, respectively, 12 months after loading (*p* = 0.047). 34% of rough-surfaced microthreaded and 56% of the machined-neck implants had 3.75 mm diameter and the rest were 4.25 mm in diameter. A positive correlation was found between an increased implant diameter and the amount of bone loss (*p* = 0.034); however, no significant differences were observed in MBL around different neck configurations according to implant diameters. Moreover, no significant differences were found in bone loss changes in terms of patient's age and gender in implant groups. Subjects were comprehensively treated with bar over dentures and fixed prostheses. The greatest marginal bone loss was attributed to machined-neck configuration in combination with bar overdentures (*p* = 0.034). They reported 98.6% survival and 97.1% success rate for machined-neck and 98.6% survival and 97.2% success rate for microthreaded implants after 12 months of loading.

Karlsson et al. [23] reported the cumulative survival rate of 97.7% for Astra Tech implants with no significant difference between machined (95.3%) and TiOblast-surfaced collars (100%) (*p* = 0.24). Moreover, the amount of bone loss did not differ significantly between the two groups, 2 years after prosthesis placement (*p* > 0.3). Another study by Van de Velde and colleagues [24] focused on implants with machined, rough, and rough microthreaded neck and followed up to one year after loading. The mean MBL was 1.52 ± 0.66 mm, 0.79 ± 0.79 mm, and 0.70 ± 1.01 mm for implants with machined, rough, and rough microthreaded neck. A significant difference in MBL existed between machined and rough neck (*p* = 0.23) and between machined and rough-surfaced microthreaded neck implants (*p* = 0.046); however, the amount of bone loss around implants with rough collars was not statistically different compared with rough microthreaded neck implants (*p* = 0.7). They reported 1-year survival rate of 98.6% for machined, 100% for rough, and 100% for rough-surfaced microthreaded neck implants.

Bratu et al. [25] found that the machined-neck implants, which showed premature exposure, exhibited significantly greater amounts of MBL compared to those with intact soft tissue coverage (*p* < 0.05). This event was not statistically significant for rough neck implants. However, it is noteworthy to mention that the low occurrence of dehiscence in both collar configurations (eight in machined and four in microthreaded neck implants) prevents the drawing of definite conclusions.

The prospective study by Shin et al. [16] compared marginal bone loss around implants with machined, rough, and rough microthreaded neck designs at 1 year after loading. Rough-surfaced microthreaded neck showed the least (0.18 ± 0.27) amount of crestal bone loss. The greatest amount of MBL was observed around machined collars which was statistically significant at every follow-up period. Neither rough collar nor rough microthreaded neck implants showed significant bone loss at 3 months after implant placement (*p* < 0.05).

### 3.3. Interinvestigator Agreement

The Kappa interinvestigator agreement was 0.87 for studies extracted from PubMed/MEDLINE, 0.86 for Embase, and 0.91 for The Cochrane Library and showed a high level of agreement.

### 3.4. Marginal Bone Loss

**Figure 2:**
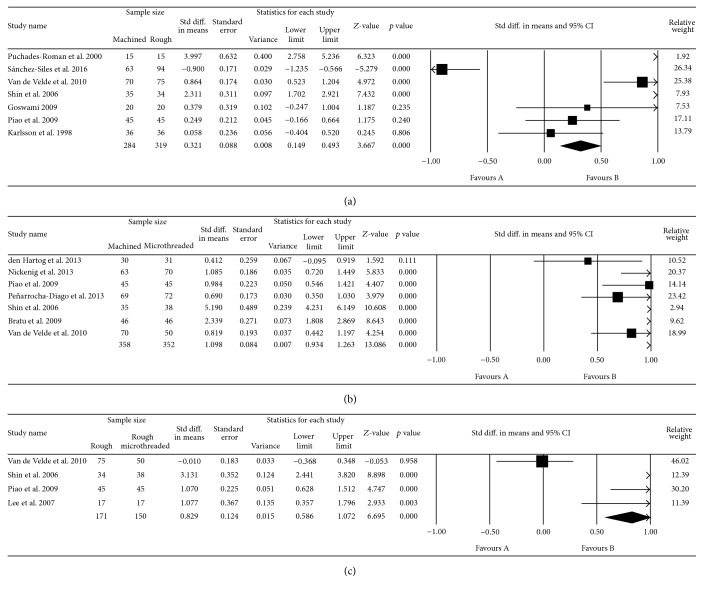
Forest plot for the event “marginal bone loss” in the comparison between machined and rough-surfaced neck implants (a), machined and rough-surfaced microthreaded neck implants (b), and rough and rough-surfaced microthreaded neck implants (c).

12 studies assessed the mean marginal bone changes (mm) around the implants in different follow-up periods. The range of marginal bone loss in machined-neck groups, rough-surfaced collar, and rough-surfaced microthreaded neck groups was 0.26 to 1.6 mm, 0.22 to 2.63 mm, and 0.14 to 0.81, respectively. Implants with rough collars showed significantly greater MBL than machined-neck implants (*p* < 0.01; MD: 0.321; and 95% CI: 0.149 to 0.493). In addition, rough-surfaced microthreaded implants had significantly higher MBL than machined (*p* < 0.01; MD: 1.098; 95% CI: 0.934 to 1.263) and rough-surfaced neck implants (*p* < 0.01; MD: 0.829; 95% CI: 0.586 to 1.072) (Figure 2).

### 3.5. Survival Rates of Implants

**Figure 3:**
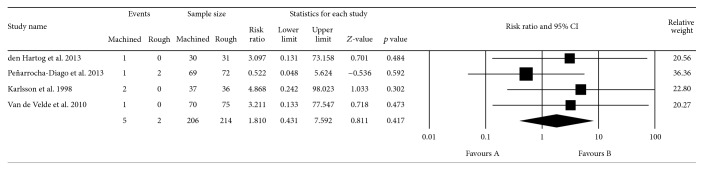
Forest plot for the event “survival rate” in the comparison between machined and rough-surfaced neck implants.

The assessed studies [3, 15, 21–25, 27] showed that 7 out of 420 implants failed (1.66%), comprising 5 machined-neck implants (1.11%) and 2 rough-surfaced microthreaded implants (0.47%) (Figure 3). All accepted studies showed more favorable survival rate in rough-surfaced implants than those with machined implants, but none showed this difference to be statistically significant. Quantitative analysis revealed that there was no statistically significant difference due to the implant neck surface characterization (*p* = 0.417; RR: 1.810; 95% CI: 0.431–7.59).

## 4. Discussion

The present systematic review showed that insertion of implants with rough and rough-surfaced microthreaded neck implants influenced the rate of bone loss and favored lesser MBL compared to machined-neck implants. Thus the null hypothesis of the study that there would be no difference between different collar surfaces of the implant with regard to marginal bone loss was rejected.

All implants have some degrees of bone loss following implant installation and loading. An early implant bone loss of 1.5 mm occurs during the healing phase and the first year in function at the crestal area of implants, followed by an annual bone loss of 0.2 mm thereafter [1, 4, 5]. Until now, the basic mechanisms underlying early peri-implant marginal bone loss are not clarified [12, 13, 20, 29, 30]. Surgical trauma, the establishment of biologic width, lack of passive fit of the superstructures, the presence of a microgap at implant-abutment interface, occlusal overload, and implant neck design are among the possible etiologic factors [2, 8, 9, 31–36].

Different implant neck designs have been proposed in order to stabilize the bone-implant contact [19, 20, 26, 29, 37]. The smooth neck implants result in reduced plaque accumulation and thus presumably prevent peri-implantitis [15, 16, 22]. However, FEA investigations revealed high stress concentrations in the area of crestal bone around the polished neck of dental implants [38, 39]. Thus MBL might be partially attributed to the lack of favorable stress distributions at the coronal portion of the implants [14].

In vivo experiments revealed that the rough-surface dental implants dramatically enhanced bone-implant interface and lowered the rate of bone loss compared with smooth surfaces [40, 41]. Moreover, the presence of microthread at the neck area might provide an increased interlocking of the implant and the marginal bone, thus reducing the MBL [14, 15, 42]. Hansson [14] found that implant surface roughness at the implant neck area leads to an increased interfacial shear strength and is effective in counteracting MBL. This result is supported by some recent clinical studies while other investigations found no significant differences in MBL [21, 23, 43, 44]. It is noteworthy to mention that the current systematic review included only those studies comparing rough or rough-surfaced microthreaded implants with machined-neck implants in order to perform a direct comparison.

The result for selected studies revealed that marginal bone changes were decreased around rough-surfaced microthreaded neck implants compared with polished and rough-surfaced neck implants. However only 2 of the 12 studies included in the meta-analysis were RCTs with greater clinical reliability. Only three studies followed up the cases more than 5 years and the rest were with 1-year follow-up. The 10-year retrospective study by Sánchez-Siles et al. [20] evaluated a total of 1244 implants with and without smooth neck and concluded that 2.5 mm smooth-necked implants suffered less bone loss and peri-implantitis at any follow-up time interval. Conversely, Chappuis et al. [29] reported a median of lower bone changes around rough implant necks after follow-up periods of 5 to 9 years. Other studies evaluated bone loss around rough necks with short follow-up periods and achieved good results [15, 19, 22, 24].

An important issue to consider is the presence of several confounding factors in the studies. It is known that titanium surface topography and chemistry affect the osseointegration [45–47]. Moderate Surface roughness improves the bone-implant contact which may have favored improved osseointegration and preservation of the marginal bone level [46]. The majority of the evaluated studies did not provide information about surface topography characterizations of implants. Implant-abutment connection is also an important factor in crestal bone level [22, 48–50]. Mostly, comparative studies were conducted among heterogeneous groups, comparing different implant neck designs and implant-abutment connections with or without platform switching with regard to marginal bone loss [15, 19, 21, 22, 24–26, 28]. Peñarrocha-Diago et al. [22] compared two groups of implants; machined-neck, externally connected, and platform matched Osseous® implants were compared with rough microthreaded, internally connected, and platform switched Inhex® implants. Greater marginal bone loss was observed in the case of Osseous implants with no platform switching. However, the possible effect of platform switching or implant-abutment connection upon marginal bone loss was not considered.

The studied population was patients with a wide range of ages. Age is an important factor that can affect bone formation and resorption. The relationship between advancing age in adults and patterns of cortical bone maintenance has been extensively documented [51, 52]. In a study by Negri et al. [53], marginal bone loss was progressively increased with age and the greatest amount of marginal bone loss was observed in women of 50 to 60 years of age.

Regarding the type of prostheses design, details of the treatment were not frequently present. Almost all of the studies rehabilitated the patients with fixed prostheses and only one was implant supported over denture. However, the effect of splinting was not defined. Splinting dissipates the loads between implants and reduces the stress and influences the results.

Data on peri-implant health were frequently not present in studies. This cannot be excluded as risk factors for peri-implant bone disease. Only Sánchez-Siles et al. [20] reported that 120 implants developed peri-implantitis and the incidence of peri-implantitis for roughened neck implants (2.92%) was much lower than smooth necks (14.41%).

There is another arguably more insidious source of confounding, however, and that is the method of assessment of MBL. Varying the X-ray exposure parameters of the different manufacturers and measurement tools may account for sources of bias. In the evaluated studies, dental intraoral, panoramic radiographs and maxillofacial CT scans provided an estimate of changes over follow-up intervals. Standardized digital intraoral radiographs were used for radiographic assessment in 10 studies. Likewise, different implant systems and neck designs were included; thus MBL could not be assessed in relation to a standard reference point. Investigating the marginal bone level changes from baseline at each follow-up time point has been suggested. However, only 7 studies reported the radiographic marginal bone changes from baseline.

Considering these limitations, the findings of the current study should be interpreted cautiously. Several other confounding factors influence the survival of implants and thus MBL is not only affected with collar configuration. Grafting, insertion of implants in freshly extracted teeth sockets, various healing periods, occlusion of the opposite arch, angulations of implants, and bone type are among other confounding variables. Prediction of these factors is only applicable when other metaregressions of two other process are performed. Study population has several confounding factors simultaneously and in this regard is considered heterogenic; hence it seems impossible to isolate risk factors as a separate study. Thus, coexistence of other risk factors in population of the study makes the evaluation of one particular risk factor impossible and the lack of control over these factors lowers the potential of definitive result extraction.

## 5. Conclusion

The result of the present systematic review revealed that marginal bone changes around rough-surfaced microthreaded neck implants were significantly lower than polished and rough-surfaced neck implants. However, considering the limitations of the current study, the results should be interpreted cautiously.

## Figures and Tables

**Table 1:** Characteristics of studies included (*n* = 12).

Author, year	Arch	Study design	Follow-up time	Patients (*n*)	Patients' age	Collar surface characterization	Implants system	Collar		Implants (*n*)	MBL (mm)			Survival rate (%)
								Diameter (mm^2^)	Length (mm)		<5 y	5–10 y	>10 y	
den Hartog et al. 2013 [3]	Maxilla	RCT	1 year	93	37.2 ± 12.9	Machined	Noble Biocare	—	1.5	30	1.19 ± 0.82	—	—	96.8
					40.1 ± 14.4	Rough microthreaded	Noble Biocare	—	—	31	0.90 ± 0.57	—	—	100
					40.1 ± 17.2	Scalloped microthreaded	Noble Biocare	—	—	31	2.01 ± 0.74	—	—	100

Nickenig et al. 2013 [19]	Mandible	RCT	5 years	34	45.2	Machined	Noble Biocare	4.3	—	63	1.3	1.4	—	—
						Rough microthreaded	Noble Biocare	4	—	70	0.6	0.7	—	—

Sánchez-Siles et al. 2016 [20]	Maxilla Mandible	Retrospective	>10 years	400	53.50	Machined	BIS Biotech	3.6/3.9/4.4	2.5	515	1.08 ± 1.27	1.12 ± 1.21	1.18 ± 1.39	—
						Rough	BIS Biotech	3.6/3.9/4.4	—	729	2.63 ± 1.61	2.39 ± 1.59	2.41 ± 1.35	—

Piao et al. 2009 [21]	Maxilla Mandible	Prospective	1 year	54	57.6	Machined	Restore	—	3	45	1.38 ± 0.71	—	—	100
						Rough	Branemark	—	—	45	1.24 ± 0.36	—	—	100
						Rough microthreaded	Hexplant	—	—	45	0.78 ± 0.49	—	—	100

Peñarrocha-Diago et al. 2013 [22]	Maxilla Mandible	Prospective	1 year	18	56.9	Machined	Osseous	3.75/4.25	—	69	0.38 ± 0.51	—	—	98.6
						Rough microthreaded	Inhex	3.75/4.25	—	72	0.12 ± 0.17	—	—	98.6

Karlsson et al. 1998 [23]	Maxilla Mandible	Prospective	2 years	50	53	Machined	Astra Tech	3.5/4	—	36	0.26 ± 0.81	—	—	95.3
						Rough	Astra Tech	3.5/4	—	36	0.22 ± 0.55	—	—	100

Van de Velde et al. 2010 [24]	Mandible	Prospective	1 year	39	58.4	Machined	Branemark	—	—	70	1.52 ± 0.64	—	—	98.6
						Rough	Astra Tech	—	—	75	0.80 ± 0.98	—	—	100
						Rough microthreaded	Astra Tech	—	—	50	0.81 ± 1.11	—	—	100

Bratu et al. 2009 [25]	Mandible	Prospective	1 year	46	23–65	Machined	MIS-Implants	3.75/4.25	—	46	1.47 ± 0.4	—	—	100
						Rough microthreaded	MIS-Implants	3.75/4.25	—	46	0.69 ± 0.25	—	—	100

Goswami 2009 [26]	Mandible	Prospective	1 year	20	25–50	Machined	Oraltronics	—	2	20	1.53 ± 0.28	—	—	—
						Rough	Nobel Biocare	—	—	20	1.41 ± 0.35	—	—	—

Shin et al. 2006 [16]	Maxilla Mandible	Prospective	1 year	68	48	Machined	Ankylos	—	—	35	1.32 ± 0.27	—	—	100
						Rough	Lifecore	—	—	34	0.76 ± 0.21	—	—	100
						Rough microthreaded	Oneplant	—	—	38	0.18 ± 0.16	—	—	100

Lee et al. 2007 [27]	Maxilla Mandible	Prospective	3 years	17	53.3	Rough microthreaded	Astra Tech	3.5/4	—	17	0.24 ± 0.13	—	—	100
						Rough	Astra Tech	3.5/4	—	17	0.51 ± 0.33	—	—	100

Puchades-Roman et al. 2000 [28]	Maxilla Mandible	Retrospective	>2 years	30	41.9	Machined	Astra Tech	—	—	15	1.65 ± (0.26)	—	—	—
					37.3	Rough	Branemark	—	—	15	0.57 ± (0.28)	—	—	—

**Table 2:** Results of quality assessment.

Quality criteria	Studies											
	den Hartog et al. [3]	Nickenig et al. [19]	Sánchez-Siles et al. [20]	Piao et al. [21]	Peñarrocha-Diago et al. [22]	Karlsson et al. [23]	Van de Velde et al. [24]	Bratu et al. [25]	Goswami [26]	Shin et al. [16]	Lee et al. [27]	Puchades-Roman et al. [28]
(1) Was the study described as random?	Yes	Yes	No	No	Yes	No	Yes	Yes	No	Yes	No	No

(2) Was the randomization scheme described and appropriate?	Yes	Yes	No	No	Yes	No	Yes	Yes	No	Yes	No	No

(3) Was the study described as double-blind?	No	No	No	No	No	No	No	No	No	No	No	No

(4) Was the method of double blinding appropriate?	No	No	No	No	No	No	No	No	No	No	No	No

(5) Was there a description of dropouts and withdrawals?	Yes	Yes	Yes	Yes	Yes	Yes	Yes	Yes	Yes	Yes	Yes	Yes

Jadad score	3	3	1	1	3	1	3	3	1	3	1	1

Quality of study	High	High	Low	Low	High	Low	High	High	Low	High	Low	Low
